# Polyfunctional T peripheral helper cells are associated with the magnitude and durability of antibody responses after COVID‐19

**DOI:** 10.1002/cti2.70070

**Published:** 2025-12-18

**Authors:** Katie Tungatt, Gabriela Martins Costa Gomes, Nicole L Fewings, Aija Stubis, Chloe M Doyle, Vera Merheb, Anupriya Aggarwal, Karen Byth, Harry Robertson, Suat Dervish, Susan Maddocks, Janette Taylor, Rowena A Bull, Marianne Martinello, the COSIN Protocol Steering Committee, Fabienne Brilot, Stuart G Turville, Anthony L Cunningham, Kerrie J Sandgren

**Affiliations:** ^1^ Centre for Virus Research The Westmead Institute for Medical Research Westmead NSW Australia; ^2^ Kids Neuroscience Centre, Kids Research Children's Hospital Westmead NSW Australia; ^3^ The Kirby Institute University of New South Wales Sydney NSW Australia; ^4^ Research and Education Network Western Sydney Local Health District Westmead NSW Australia; ^5^ School of Mathematics and Statistics University of Sydney Sydney NSW Australia; ^6^ Sydney Precision Data Science Centre University of Sydney Sydney NSW Australia; ^7^ Centre for Transplant and Renal Research The Westmead Institute for Medical Research Westmead NSW Australia; ^8^ Westmead Cytometry, Westmead Research Hub The Westmead Institute for Medical Research Westmead NSW Australia; ^9^ Westmead Hospital Westmead NSW Australia; ^10^ School of Medical Sciences, Faculty of Medicine and Health The University of Sydney Sydney NSW Australia; ^11^ Centre for Infectious Diseases and Microbiology Laboratory Services Institute of Clinical Pathology and Medical Research, Westmead Hospital Westmead NSW Australia; ^12^ School of Biomedical Sciences Faculty of Medicine & Health, University of New South Wales Sydney NSW Australia; ^13^ Blacktown Hospital Blacktown NSW Australia; ^14^ Prince of Wales Hospital Randwick NSW Australia; ^15^ Brain and Mind Centre University of Sydney Sydney NSW Australia; ^16^ Sydney Infectious Diseases Institute (SydneyID) Faculty of Medicine and Health, The University of Sydney Sydney NSW Australia; ^17^ Present address: School of Medicine and Public Health The University of Newcastle Newcastle NSW Australia

**Keywords:** age, antibody levels, COVID‐19, IL‐21, peripheral helper T cells, polyfunctional T cells

## Abstract

**Objective:**

Examine the role of T cells in shaping and sustaining humoral immunity to SARS‐CoV‐2 and identify immune factors associated with durable antibody responses.

**Methods:**

Unvaccinated adults (*n* = 67) with SARS‐CoV‐2 infection (Wuhan) of any severity were followed longitudinally for up to 14 months. Anti‐Spike (S) binding and neutralising antibodies were measured. Bulk T cell IFNγ and IL‐2 responses were assessed by Fluorospot to multiple viral proteins. S‐specific T‐cell subsets and functions were analysed in detail by flow cytometry in a subset of participants (*n* = 14), combining activation‐induced markers (AIMs) and cytokines. Correlations between S‐specific T‐cell subsets, their polyfunctionality and antibody levels over time were defined.

**Results:**

Over 14 months post infection, anti‐S IgG and neutralising antibodies declined significantly but remained detectable in > 85% of participants. Bulk T‐cell IFNγ and IL‐2 responses persisted without significant reduction. However, S‐specific CD4 T cells declined over time. A large proportion of these were peripheral helper T cells (Tph; CXCR5^−^PD‐1^+^), which were the main producers of IL‐21 and showed marked polyfunctionality during early convalescence, in contrast to circulating follicular helper T cells (cTfh). The early presence of polyfunctional, IL‐21‐producing Tph strongly correlated with S‐specific IgG and neutralising antibodies early post‐infection and predicted neutralising antibody maintenance a year later. Older age correlated with higher Tph and antibody levels.

**Conclusion:**

Early S‐specific T‐cell responses—particularly polyfunctional, IL‐21‐producing Tph cells—play a key role in sustaining durable antibody levels post‐infection, offering insights for future vaccine strategies and understanding long‐term immunity.

## Graphical abstract

Early polyfunctional T peripheral helper (Tph) cells producing IL‐21 after SARS‐CoV‐2 infection correlate with early neutralising antibodies and their long‐term persistence. Tph and neutralising antibody levels increased with age. These findings highlight the crucial role of Tph cells in sustaining SARS‐CoV‐2 humoral immunity and offer insights for future vaccine strategies.

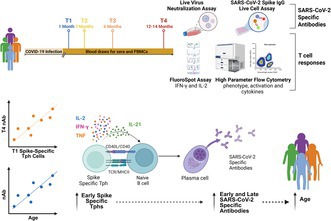

## Introduction

The COVID‐19 pandemic has highlighted the importance of longitudinal immune studies to understand long‐term viral immunity. Understanding the dynamics of humoral and cellular immunity is crucial for characterising the durability and breadth of T‐cell and B‐cell memory responses following infection and vaccination. This knowledge is key to determining how immunological memory affects disease severity, reinfection risk by emerging viral variants and effectiveness of hybrid immunity and will inform treatment and vaccination strategies.

Host factors including age, comorbidities, disease severity and vaccination status influence the magnitude and kinetics of protective antibodies and cellular immune responses.^1^, ^2^, ^3^ Interactions between these immune components are also critical for robust and long‐lasting immunity. CD4^+^ T‐cell subsets, particularly T peripheral helper (Tph) and T follicular helper (Tfh) cells, contribute significantly to antibody production and B‐cell memory formation and the duration of these responses.^4^, ^5^, ^6^

Robust antibody responses are crucial for viral clearance during COVID‐19. Following SARS‐CoV‐2 infection, IgG antibodies typically peak within 2–4 weeks, while neutralising antibodies peak around 3–4 weeks.^7^, ^8^ However, antibodies wane significantly over time, typically with a bi‐exponential pattern characterised by a rapid decline around 30–50 days post symptom onset, followed by a more gradual decrease.^9^, ^10^, ^11^ Neutralising antibodies decrease significantly by 90 days post symptom onset^8^, ^12^ although IgG titres stabilise after several months.^11^, ^13^, ^14^, ^15^ Disease severity and age significantly influence these dynamics: Severe infections produce higher antibody levels^10^, ^16^, ^17^ and older age correlates with higher antibody levels and longer half‐lives.^16^, ^18^, ^19^, ^20^

SARS‐CoV‐2 infection also induces robust CD4^+^ and CD8^+^ T‐cell responses, which persist for at least 6 months.^5^, ^9^, ^12^, ^13^, ^21^, ^22^ There is a qualitative shift in SARS‐CoV‐2‐specific CD4^+^ T cells over time: initially producing a mixture of interferon‐gamma (IFNγ), interleukin 2 (IL‐2) and tumor necrosis factor (TNF), which subsequently transitions to cells producing only TNF.^23^ Polyfunctional CD4^+^ T cells co‐expressing IFNγ, IL‐2, and TNF are the dominant subset in non‐hospitalised individuals, peaking around 6 months post recovery and the frequency of these polyfunctional CD4^+^ T cells correlates with viral control.^24^ Various CD4^+^ T‐cell subsets contribute to this response: activated circulating T follicular helper (cTfh) cells appear during acute infection, with memory cTfh cells supporting sustained antibody production.^4^, ^12^, ^17^, ^25^ Tph cells (PD‐1^+^CXCR5^−^CD4^+^) are lso elevated in SARS‐CoV‐2, characterising early immune responses and have been associated with plasmablast differentiation and atypical B‐cell activation.^6^, ^26^

There are still critical knowledge gaps regarding how distinct CD4^+^ T‐cell subsets govern the persistence and functional maturation of antibody responses over time.^4^, ^5^, ^17^, ^27^ Although there is clear evidence that CD4^+^ T cells, particularly Tfh subsets in the lymph node, are crucial for the generation and long‐term maintenance of neutralising antibody,^1^, ^12^, ^17^, ^28^ the precise mechanisms and kinetics governing this interdependence are not yet fully elucidated.^4^, ^28^ Specifically, the direct relationship between the quality of memory CD4^+^ T cells—including cytokine profile, polyfunctionality and subset balance—and the persistence or functional maturation of memory B cell and antibody responses over time remains inadequately defined.^4^, ^23^, ^28^ The functional quality and role of regulatory T cells (Tregs), cTfh and Tph in long‐term humoral immunity, beyond 6 months, require further research.^4^, ^6^, ^29^ The influence of age and disease severity on CD4^+^ T‐cell subset functionality and long‐term quality also remains incompletely characterised over extended timeframes.

This lack of understanding impedes the development of optimal vaccine booster strategies and the accurate prediction of long‐term protection against emerging viral variants. Therefore, this study aimed to analyse antibody and T‐cell memory responses over 12 months among people infected with the original Wuhan strain of SARS‐CoV‐2, presenting with varying disease severity. We employed a novel, integrated approach combining activation‐induced marker (AIM) measurements with intracellular cytokine staining (ICS) in the same sample to characterise T‐cell polyfunctionality at a subset level, including cTfh and Tph, and its correlation with antibody levels over the 12 months. Notably, our study uncovered unexpected direct correlations between age, sustained antibody responses and polyfunctional T‐cell subsets, including IL‐21 production. These results offer a crucial comparison to vaccine responses, complementing previous shorter term studies and those with a narrower or partially overlapping range of parameters.

## Results

This analysis included 67 participants with laboratory‐confirmed SARS‐CoV‐2 infection followed longitudinally for up to 14 months (range 1–14 months) (Table 1). Participants did not receive neutralising monoclonal antibody therapy and were not vaccinated or re‐infected with SARS‐CoV‐2 during the follow‐up period. The cohort was evenly split into three age groups: 24–59, 60–69 and ≥ 70 years with a median age of 62 years (range 24–85; IQR 51, 71). The oldest age group was selected to reflect the increased risk of severe COVID‐19 after age 70, which aligns with a decline in immune function, while the 60–69 age group served as a transitional phase.^12^ In our cohort however, most participants had infection of mild (56.7%) or moderate (28.3%) severity; 12% had severe infection, of whom only 3% were aged ≥ 70 years. We assessed binding and neutralising antibodies, along with T‐cell responses, at four time points: 1 month (T1; range 1–3 months), 3 months (T2; range 3–6 months), 6 months (T3; range 6–9 months) and 12 months (T4; range 11–14 months).

**Table 1:** Cohort demographics

Age group (years old)	24–59	60–69	70+	Total cohort
*n* (%)	26 (38.8%)	21 (31.3%)	20 (29.9%)	67
Sex				
Male	13 (50.0%)	11 (52.4%)	10 (50.0%)	34 (50.7%)
Female	13 (50. 0%)	10 (47.6%)	10 (50.0%)	33 (49.3%)
Disease severity				
Mild	15 (57.7%)	10 (47.6%)	13 (65.0%)	38 (56.7%)
Moderate	7 (26.9%)	7 (33.3%)	5 (25.0%)	19 (28.3%)
Severe	2 (7.7%)	1 (4.8%)	1 (5.0%)	4 (6.0%)
Critical	1 (3.8%)	2 (9.5%)	1 (5.0%)	4 (6.0%)
Unknown	1 (3.8%)	1 (4.8%)	0 (0.0%)	2 (3.0%)

### Antibody levels declined but persisted up to 14 months after SARS‐CoV‐2 infection

**Figure 1:**
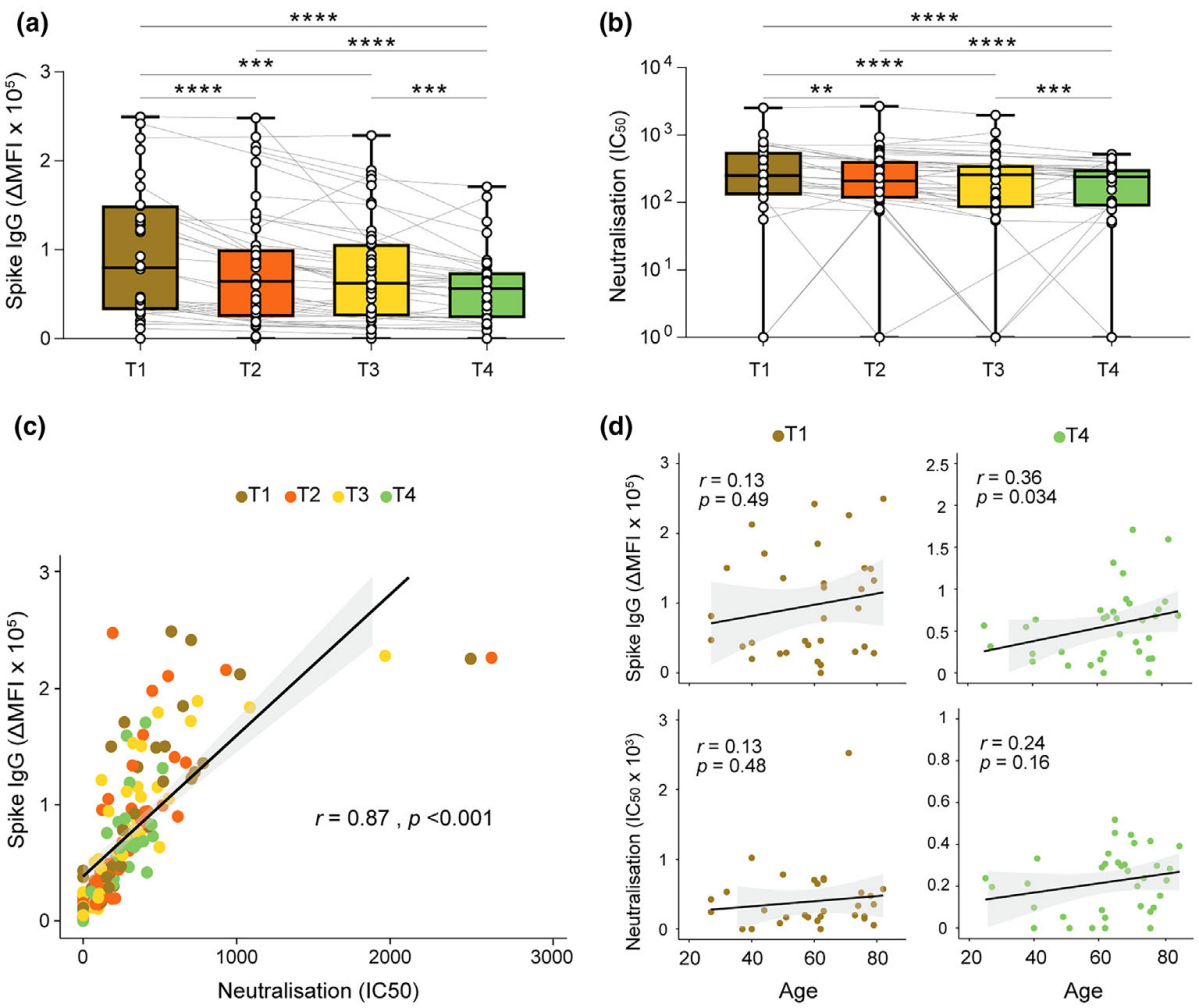
Anti‐SARS‐CoV‐2 spike IgG and neutralising antibody titres post SARS‐CoV‐2 infection. **(a)** Anti spike IgG (measured by delta median fluorescence intensity (∆MFI)) and **(b)** virus neutralisation against the original Wuhan strain were measured, where available, at four timepoints. Timepoint 1, 2, 3 and 4 (T1, T2, T3, T4) were collected post infection at ~1–3 months, ~3–6 months, ~6–9 months and ~11–14 months, respectively. Box plots display the median and interquartile range. Mixed effects analysis was performed with **P ≤ 0.01; ***P ≤ 0.001; ****P ≤ 0.0001. **(c)** Correlation between anti spike IgG and neutralising antibodies for all available data. Time points are colour‐coded. Spearman's rank coefficient (*r*) and *P*‐value are displayed inset. **(d)** Correlation between anti spike IgG or neutralising antibodies and participant age at virus infection for T1 and T4. Spearman's rank coefficient and *P*‐value are displayed inset.

Natural SARS‐CoV‐2 infection triggers the production of both non‐neutralising and neutralising antibodies, essential for clearing the virus and ensuring long‐term protection against infections and diseases.^9^, ^20^ At T1, anti‐S IgG and neutralising activity against the ancestral strain were detectable in most participants (96.9% and 84.4%, respectively) and remained detectable at T4 (94.3% and 85.7%, respectively). Anti‐S IgG and neutralising titres showed a significant and progressive decline across all time points and on a continuous scale of months post‐infection, although there was marked individual variability within each category of disease severity (Figure 1a and b, Supplementary figure 1, Supplementary table 1). The rate of antibody waning decreased over time from diagnosis to sample collection (*P* < 0.001 linear time covariate and *P* = 0.001 quadratic time covariate) (Supplementary table 1). However, the data best fit a model of constant decay (IgG *t*_1/2_: 431 days and neutralising antibody *t*_1/2_: 350 days). This decay was independent of age (Supplementary table 1). Anti‐S IgG and neutralisation activity were strongly correlated at all time points (*r* = 0.87, *P* < 0.001), as shown in Figure 1c and previously described.^20^ Advancing age was correlated with higher anti‐S IgG levels at T4, 12 months post infection (Figure 1d, *r* = 0.36, *P* = 0.034), although not with neutralising titre levels. Similarly, no significant correlations between age and either anti‐S IgG or neutralisation titre were observed at earlier time points in this cohort, although positive trends were noted (Figure 1d). Linear mixed effects (LME) modelling indicated that the rates of decline for both anti‐S IgG and neutralising titre were not significantly affected by age group (Supplementary table 1).

### IFNγ and IL‐2 T‐cell responses persisted for 12 months following SARS‐CoV‐2 infection

**Figure 2:**
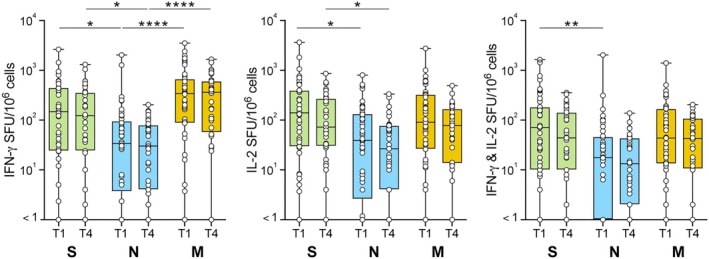
T‐cell responses to spike (S), nucleocapsid (N) and membrane (M) peptide pools post SARS‐CoV‐2 infection. Total IFN‐y, total IL‐2 and dual positive IFN‐y and IL‐2 cells were detected by fluorospot following 20‐h stimulation with Miltenyi SARS‐CoV‐2 S, N or M peptide pools. Responses were measured from 1 to 14 months post infection. Time point 1 and time point 4 (T1 and T4) are displayed. Data were background subtracted and are displayed as spot‐forming units (SFU) per 10^6^ PBMC. Box plots display the median and interquartile range. Kruskal–Wallis tests with Dunn's multiple comparisons were performed with *P < 0.05; **P ≤ 0.01; ****P ≤ 0.0001.

**Figure 3:**
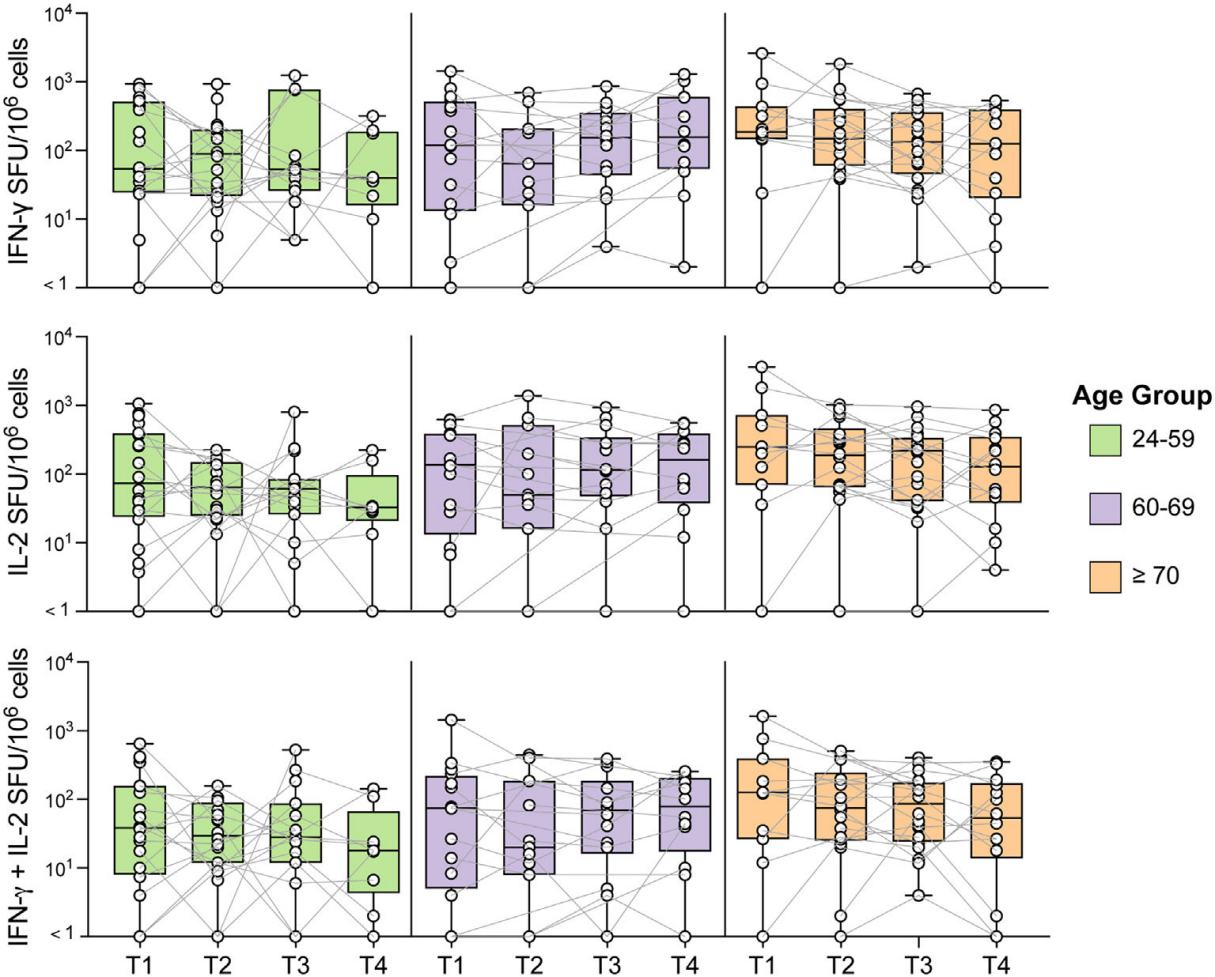
Longitudinal T‐cell responses to spike peptide pool by age group after post‐SARS‐CoV‐2 infection. Total IFN‐γ, total IL‐2, and dual‐positive IFN‐γ and IL‐2 cells were detected by fluorospot following 20 h of stimulation with Miltenyi spike peptide pool. Responses were measured from 1 to 14 months post‐infection across four time points (T1, T2, T3, and T4). Data were background‐subtracted and are displayed as spot‐forming units (SFU) per 10^6^ PBMC. Box plots display the median and interquartile Range. Data are split by age at the time of infection: 24–59, 60–69 and ≥70 years old. Linear mixed‐effects modelling was performed on log‐transformed data and is reported in Supplementary table 1, with no significant effects of time or age on any T‐cell parameter.

T‐cell responses were also assessed up to 14 months post infection. Previous studies have shown that the structural proteins S, N and M are immunodominant in individuals with SARS‐CoV‐2 infection, eliciting strong responses in CD4 and CD8 T cells.^30^ PBMC samples were therefore stimulated *in vitro* with overlapping peptide pools of the S, N and M proteins from the SARS‐CoV‐2 ancestral strain and T‐cell cytokine release was measured by FluoroSpot. Following SARS‐CoV‐2 infection, most participants exhibited detectable IFNγ, IL‐2, and dual IFNγ and IL‐2‐producing cells in response to at least one of these SARS‐CoV‐2 proteins. At T1, 85.1%, 68.9% and 90.7% of participants showed cytokine responses to S, N and M proteins, respectively. The S and M pools elicited the most robust cytokine responses, generating significantly higher IFNγ levels than the N pool at both the T1 and T4 time points. Furthermore, S pool stimulation resulted in significantly higher IL‐2 and dual‐producing cell responses than N at T1, with the difference in IL‐2 response persisting at T4 (Figure 2). LME models revealed that T‐cell cytokine responses to S, N and M peptide pools did not wane post‐infection and were not influenced by age group (Figure 3, Supplementary table 1).

### T‐cell subset dynamics post‐COVID‐19 infection

To gain a comprehensive understanding of T‐cell responses to the S protein, which is the most relevant for B:T cell interactions to generate neutralising antibodies^21^, we used high‐parameter flow cytometry to simultaneously analyse various T‐cell subsets, AIMs and cytokine production in response to a whole S peptide pool. We conducted paired data comparisons for early (T1) and late (T4) time points post‐infection on a subset of 14 participants with available samples, all of whom had mild or moderate disease. This cohort was sex‐balanced and distributed across the three age groups (Supplementary table 2).

The proportions of total CD4 and CD8 T cells remained stable between T1 and T4 (Supplementary figure 3). While the overall frequency of the FoxP3^+^ Treg population remained stable between T1 and T4, significant changes were noted within Treg subsets (Supplementary figure 4). Notably, the proportion of mature CD45RO^+^CD39^+^ Tregs significantly decreased at T4 compared to T1 (*P* ≤ 0.001). In contrast, the percentages of both naïve CD45RO^−^CD39^−^ and mature CD45RO^+^CD39^−^ Tregs significantly increased over the same period (*P* ≤ 0.01 for both), indicating a compositional restructuring of the Treg pool during late convalescence. This restructuring suggests a shift toward a less suppressive phenotype, given that CD45RO^+^CD39^+^ Tregs are known to be highly suppressive^31^, ^32^ as the immune response to SARS‐CoV‐2 resolves infection.

**Figure 4:**
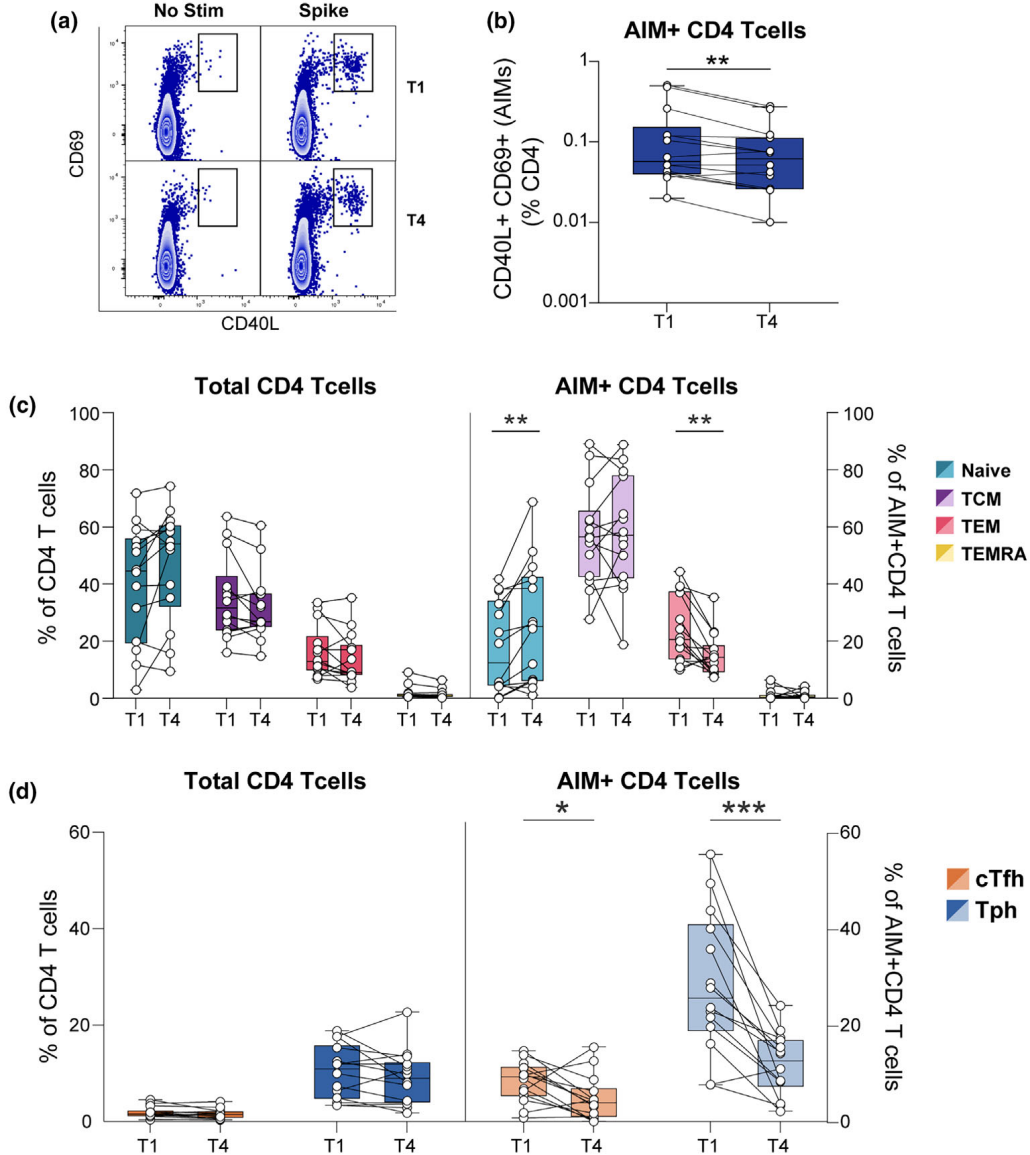
Longitudinal analysis of CD4 T‐cell populations following SARS‐CoV‐2 infection. The frequency and phenotype of CD4 T cells, including both total and spike‐specific populations, were assessed at two time points: 1–3 months (T1) and 11–14 months (T4) post‐infection. **(a)** Flow cytometry plots are presented to show AIM^+^ SARS‐CoV‐2‐specific CD4 T cells (CD40L^+^CD69^+^CD4 T cells) following a 6‐h stimulation with a spike peptide pool, in contrast to unstimulated conditions. **(b)** The frequency of AIM^+^ CD4 T cells, adjusted for background, is reported as a percentage of CD4^+^ T cells. **(c)** The figure illustrates changes in the frequency of CD4 T cell subsets within both total CD4 T cells (left) and AIM^+^ CD4 T cells (right), including naïve, central memory (TCM), effector memory (TEM), and effector memory T cells re‐expressing CD45RA (TEMRA). **(d)** Changes in the frequency of circulating follicular helper T cells (cTfh, CD4^+^CXCR5^+^ PD‐1^+^) and peripheral helper T cells (Tph, CD4^+^CXCR5^−^PD‐1^+^) within total CD4 T cells (left) and AIM^+^ CD4 T cells (right). Statistical significance was evaluated using the Wilcoxon matched‐pair signed‐rank test. *P < 0.05, **P ≤ 0.01, ***P ≤ 0.001.

SARS‐CoV‐2 S‐specific CD4 T cells were identified via an AIM assay, defined by the co‐expression of CD40L and CD69 following stimulation with a whole S peptide pool (Figure 4a). A significant decrease in the frequency of S‐specific, AIM^+^ CD4 T cells was observed between the T1 and T4 time points (*P* ≤ 0.01, Figure 4b), consistent with the expected contraction phase following an acute response.^12^ Next, we assessed the memory phenotype of both total and S‐specific CD4 T cells. Analysis of the total CD4 T cell compartment showed no significant longitudinal changes in the proportions of naïve (CCR7^+^CD45RO^−^), central memory (TCM; CCR7^+^CD45RO^+^), effector memory (TEM; CCR7^−^ CD45RO^+^) or terminally differentiated effector memory re‐expressing CD45RA (TEMRA; CCR7^−^CD45RO^−^) subsets between T1 and T4 (Figure 4c, left panel). In contrast, the phenotype of the S‐specific, AIM^+^ CD4 T cells changed significantly over time. Within the AIM^+^ population, the proportion of naïve CD4 T cells increased (*P* ≤ 0.01), while the proportion of TEM cells significantly decreased (*P* ≤ 0.01) from T1 to T4 (Figure 4c, right panel). We also examined two populations important for providing T cell help to antibody responses: cTfh (CD4^+^CXCR5^+^PD‐1^+^) and Tph (CD4^+^CXCR5^−^PD‐1^+^).^33^ Within the total CD4 T‐cell pool, the frequencies of cTfh and Tph remained stable between T1 and T4 (Figure 4d, left panel); however, S‐specific, AIM^+^cTfh (*P* < 0.05) and Tph (*P* ≤ 0.001) significantly decreased from T1 to T4 (Figure 4d, right panel). Thus, these specific helper subsets waned within the antigen‐specific pool post‐infection.

We also looked at total CD8 T‐cell proportions of naïve, TCM, TEM and TEMRA subsets between T1 and T4, finding a significant decrease in TCM over time post‐infection (Supplementary figure 5). In contrast to S‐specific CD4 T‐cell responses, we detected minimal S‐specific AIM^+^ CD8 T cells, defined here by co‐expression of activation markers CD107a and CD69, with no significant change in their proportion from T1 to T4 (Supplementary figure 6). This likely reflects that the S peptide pool is targeted mainly towards CD4 T cells with 15‐mer peptides, although some internal CD8 T‐cell epitopes are present.

### Spike (S)‐specific CD4 T cells exhibited reduced polyfunctionality over time

**Figure 5:**
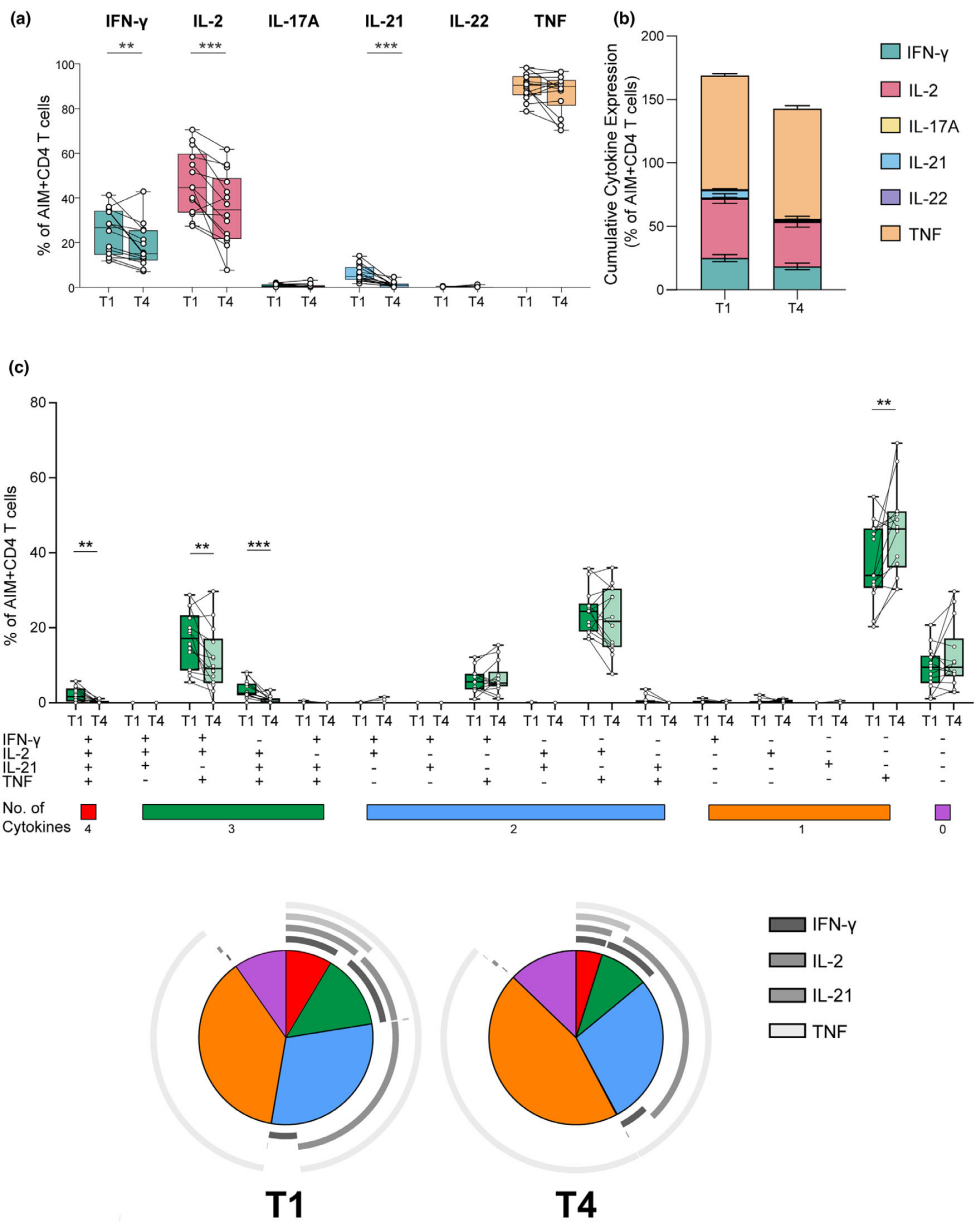
Polyfunctionality of spike‐specific CD4 T cells over time following SARS‐CoV‐2 infection. The proportions of AIM^+^ CD4 T cells producing cytokines IFN‐γ, IL‐2, IL‐21, and TNF were measured by flow cytometry at T1 (1–3 months) and T4 (11–14 months) post‐infection. **(a)** The percentage of total cytokine‐producing AIM^+^ CD4 T cells is shown at T1 and T4. **(b)** Cumulative cytokine expression, including polyfunctional CD4 T cells. **(c)** The proportions of polyfunctional AIM^+^ CD4 T cells, producing various combinations of cytokines. The coloured bars at the bottom of the bar graph indicate the degree of polyfunctionality. Pie charts display the proportions of different cytokine‐producing AIM^+^ CD4 T‐cell combinations at time points T1 and T4. The colours of the wedges represent the degree of polyfunctionality, as mentioned above, and the pie arches indicate the specific cytokine combinations produced in each wedge. Statistical significance was assessed using the Wilcoxon matched‐pairs signed rank test. **P ≤ 0.01, ***P ≤ 0.001.

In this study, we integrated the analysis of AIMs and cytokine production within the same cells, which are typically reported separately. AIM^+^ CD4 T cells release cytokines in response to S peptides, notably IFNγ, IL‐2, TNF and IL‐21. All these cytokines showed a significant decrease from T1 to T4, except for TNF, which remained elevated at T4 (Figure 5a and b). Furthermore, the polyfunctionality of the cytokine‐producing S‐specific CD4 T cells was characterised: A small fraction (median 1.65%) of these cells produced all four cytokines at T1, but they were rare at T4 (Figure 5c). Triple cytokine‐producing cells were either IFNγ^+^IL‐2^+^TNF+ (median 17.19% at T1) or IL‐21^+^IL‐2^+^TNF^+^ (median 2.50% at T1); these populations significantly decreased over time to medians of 9.15% and 0.82%, respectively, at T4. TNF was the predominant cytokine detected, with most S‐specific CD4 T cells producing either TNF alone or in combination with IL‐2. The latter remained stable over time (T1 median 24.39% and T4 median 21.70%), and T cells retained the ability to produce TNF even as their other functions diminished. Thus, single cytokine TNF‐producing cells significantly increased from T1 (median 33.98%) to T4 (median 46.37%). In contrast, IL‐21 production was almost gone by T4. In summary, while IFNγ and IL‐2 production from bulk T cells, as measured by Fluorospot, was maintained over time post‐infection in the complete cohort (Figure 3), a more detailed examination of S‐specific CD4^+^ T cells by ICS revealed subset and polyfunctional (3–4 cytokine) responses that declined over time (Figure 5c).

### Cytokine profiling identifies Tph‐like cells as major producers of IL‐21 and highly polyfunctional early post‐SARS‐CoV‐2 infection

**Figure 6:**
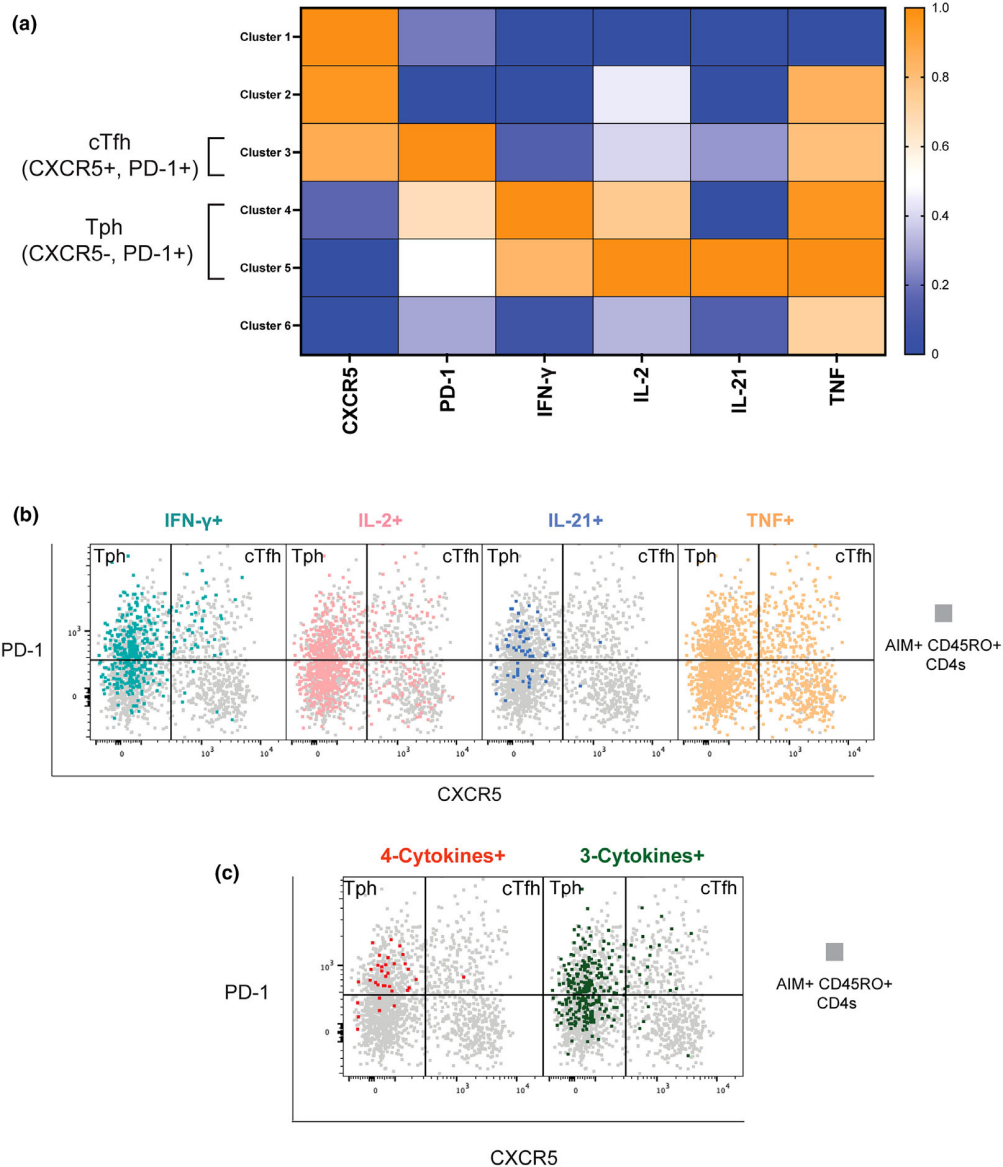
Cluster analysis reveals cytokine profiles of cTfh and Tph AIM^+^ memory CD4 T cells. **(a)** Heatmap depicting FlowSOM clustering results for activated (AIM^+^) CD45RO^+^ memory CD4 T cells at T1 (1–3months) post‐infection. Normalised mean fluorescence intensity (MFI) is shown for phenotypic markers (CXCR5, PD‐1) and intracellular cytokines (IFNγ, IL‐2, IL‐21, TNF) across six identified clusters. Clusters were manually annotated as circulating T follicular helper (cTfh)‐like (Clusters 1–3; CXCR5^+^, PD‐1^+^) or T peripheral helper (Tph)‐like (Clusters 4–6; CXCR5^−^, PD‐1^+^). MFI values were scaled from 0 (blue) to 1 (orange). **(b)** Representative flow cytometry overlays showing PD‐1 versus CXCR5 expression on total AIM^+^ memory CD4 T cells (AIM^+^ CD45RO^+^; grey). Cells producing specific cytokines upon stimulation are overlaid: IFNγ^+^ (teal), IL‐2^+^ (pink), IL‐21^+^ (blue), and TNF^+^ (orange). Quadrant gates delineate Tph‐like (CXCR5^−^, PD‐1^+^) and cTfh‐like (CXCR5^+^, PD‐1^+^) populations. **(c)** Representative flow cytometry overlays illustrating the distribution of polyfunctional T cells within the PD‐1 and CXCR5 expression space. Cells co‐expressing 4‐cytokines (red) or any 3‐cytokines (green) are overlaid onto the total AIM^+^ memory CD4 T cell population (AIM^+^ CD45RO^+^; grey). Quadrant gates are consistent with panel B.

To characterise the functional profiles of SARS‐CoV‐2‐specific memory CD4 T‐cell subsets during early convalescence, we performed unsupervised clustering analysis on AIM^+^ CD4 T cells obtained 1–3 months post infection (T1). Individual samples stimulated with S peptides were concatenated together for this analysis, and clustering was based on the expression of phenotypic markers (CXCR5, PD‐1) and intracellular cytokines (IFNγ, IL‐2, IL‐21, TNF). From clustering analysis, we identified six distinct cell clusters corresponding to specific cytokine signatures as revealed by the normalised mean fluorescence intensity (MFI) heatmap. Among these clusters, three were manually annotated as either cTfh (Clusters 3; CXCR5^+^PD‐1^+^) or Tph (Clusters 4–5; CXCR5^−^PD‐1^+^) cells (Figure 6a). The Tph clusters displayed prominent expression profiles for multiple cytokines, including IFNγ, IL‐2, IL‐21 and TNF, compared to the cTfh cluster (Figure 6a).

To further investigate the contribution of these subsets to specific cytokine responses, we visualised the expression of individual cytokines on AIM^+^CD45RO^+^ populations by looking at PD‐1 vs CXCR5 plots. The concatenated cohort flow cytometry overlays demonstrated that IFNγ (teal) and IL‐21 (blue)‐producing cells were abundant within the Tph gate (CXCR5^−^PD‐1^+^), compared to the cTfh gate (CXCR5^+^PD‐1^+^) (Figure 6b). TNF (orange) and IL‐2 (pink)‐producing cells were observed in both compartments, with IL‐2 appearing more prevalent within the Tph subset (Figure 6b).

Given the important role of polyfunctional T cells in effective immunity, we visualised cells producing either any three cytokines (green) or all four cytokines (red) onto the total AIM^+^CD45RO^+^CD4 T cell population, revealing that these highly polyfunctional cells were predominantly located within the Tph (CXCR5^−^PD‐1^+^) gate (Figure 6c). Together, these data indicate that at 1–3 months post SARS‐CoV‐2 infection, S‐specific Tph memory CD4 T cells represent a significant source of the B‐cell helper cytokine IL‐21 and exhibit the highest degree of polyfunctionality compared to their cTfh counterparts.

### Spike (S)‐specific CD4 T‐cell populations tightly correlated with antibody levels

**Figure 7:**
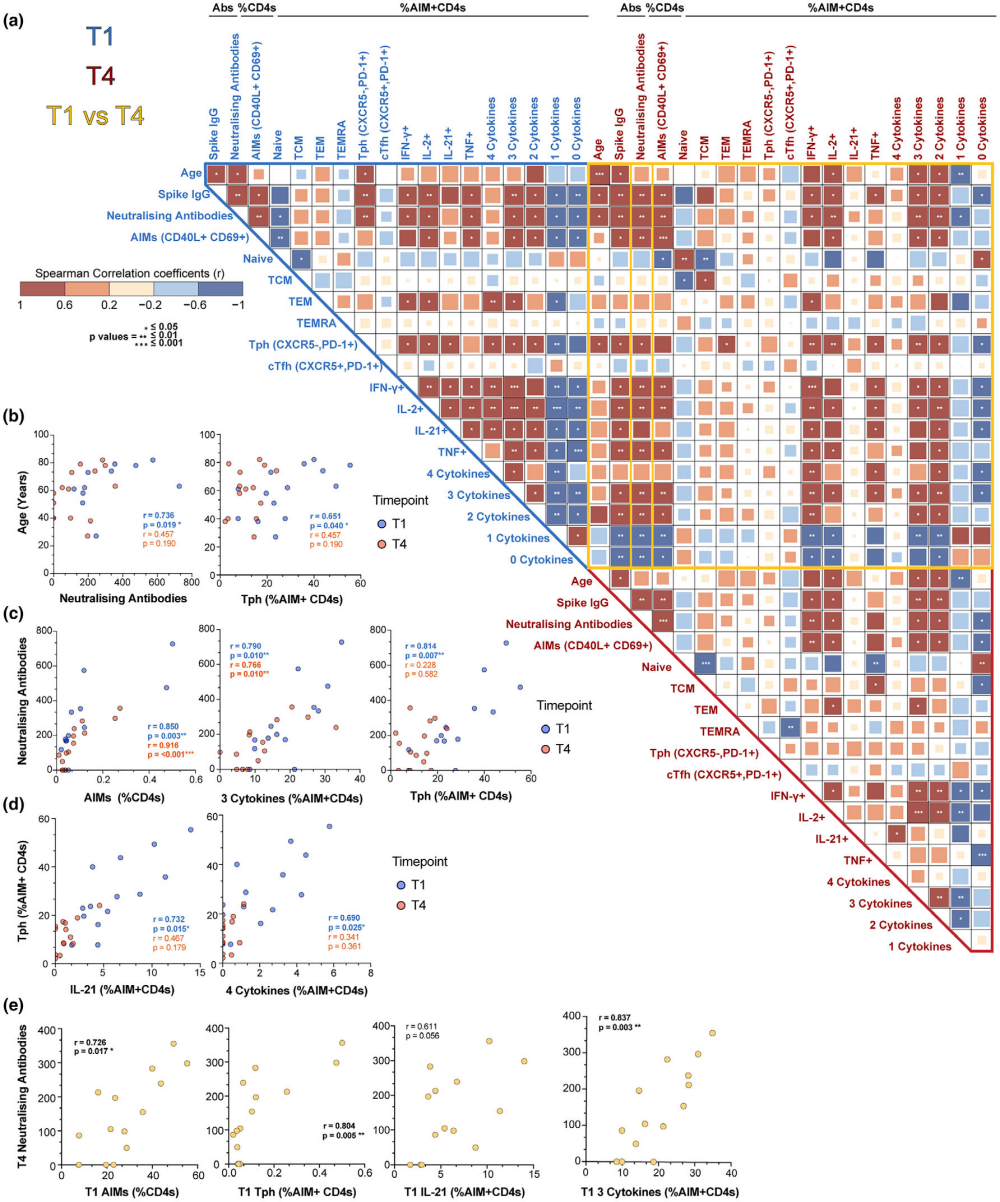
Association of age, antibodies and AIM^+^ CD4 T cells at different timepoints post SARS‐CoV‐2 infection. **(a)** Correlation matrix heat map of multiple parameters at T1 (1–3 months) and T4 (11–14 months) post infection. Spearman's rank correlation coefficients (*r*) are depicted by colour intensity, ranging from blue (negative correlation, *r* = −1.0) to red (positive correlation, *r* = 1.0), and the size of the squares. Statistical significance (*P*‐values adjusted for multiple comparisons using the Benjamini–Hochberg (BH) method) is indicated by asterisks: *P < 0.05, **P ≤ 0.01, ***P ≤ 0.001. Borders indicate the comparison type: blue border = T1 vs T1 correlations; yellow border = T1 vs T4 correlations; red border = T4 vs T4 correlations. B–D) Correlation scatter plots of parameters of interest. Dots and statistics for T1 are in blue and red for T4. **(b)** Age vs neutralising antibodies and Tph (CXCR5^−^PD‐1^+^, % AIM^+^ CD4 T cells); **(c)** Neutralising antibodies vs AIM^+^ CD4 T cells, polyfunctional (3 cytokines) AIM^+^ CD4 T cells, Tph. **(d)** Tph vs IL‐21, polyfunctional (4 cytokines) AIM^+^ CD4 T cells **(e)** T4 neutralising antibodies vs T1 AIM^+^ CD4 T cells, Tph, IL‐21, polyfunctional (3 cytokines) AIM^+^ CD4 T cells.

To gain a clearer understanding of the relationship between S‐specific CD4 T cell subsets and their functions in conjunction with various other parameters, a Spearman' correlation matrix was conducted using key T‐cell subsets from the flow cytometry dataset, along with time post‐infection, age, IgG, and neutralising titres for both early convalescence (T1) and 11–14 months post infection (T4) (Figure 7a). In this cohort of participants with mild or moderate disease, a strong positive correlation was observed between S‐binding IgG and neutralising titres at 1–3 months post infection (T1) and was maintained at 11–14 months (T4), aligning with previous findings (Figure 7a). Furthermore, a significant positive correlation emerged between both anti‐S IgG and neutralising titre levels and advancing age at T1, an association that was maintained for anti‐S IgG at T4. Age also positively correlated with the frequency of S‐specific AIM^+^ Tph cells (CXCR5^−^PD‐1^+^) at T1 (Figure 7b).

Among total T‐cell subsets, the only correlation with antibody characteristics was a significant negative association between naïve Tregs (CD45RO^−^, CD39^−^) and anti‐S‐gG and neutralising titres during the early convalescence phase (T1); however, this correlation was not observed at the later time point T4 (Supplementary figure 7). In the early convalescence phase, both anti‐S and neutralising titre levels strongly correlated with the frequency of S‐specific AIM^+^ and polyfunctional CD4 T cells, specifically those co‐expressing three cytokines and the AIM^+^ Tph cell subset (Figure 7c). Within the early cellular response, the frequency of these AIM^+^ Tph cells was itself positively correlated with CD4 T cells producing IL‐21 and producing four cytokines (IFNγ, IL‐2, TNF and IL‐21) (Figure 7d). Importantly, analysis of longitudinal relationships revealed that the maintenance of neutralising titres at T4 strongly correlated with the magnitude of several S‐specific cellular responses present during early convalescence (T1), including total AIM^+^ CD4 T cells, AIM^+^ Tph cells, IL‐21‐producing AIM^+^ cells, and 3‐cytokine polyfunctional AIM^+^ CD4 T cells (Figure 7e), underscoring the predictive capacity of early T‐cell responses for durable humoral immunity. In this cohort, the positive relationship between these early T‐cell responses and late convalescence neutralising antibody levels was also associated with advancing age.

## Discussion

The SARS‐CoV‐2 pandemic highlighted the need for comprehensive longitudinal studies to gain insight into the complexity of adaptive immune responses post‐infection, particularly regarding T‐ and B‐cell memory durability. This study characterised humoral and cellular immunity in 67 unvaccinated adults from Sydney, Australia, for up to 14 months following predominantly mild‐to‐moderate SARS‐CoV‐2 infection during the initial Wuhan variant wave. A key strength was the novel combination of AIM measurements and intracellular cytokine staining in the same sample, enabling characterisation of S‐specific T‐cell polyfunctionality at the subset level, alongside correlations with antibody levels. While a persistent, albeit declining, antibody response was observed alongside stable bulk T‐cell reactivity, there was a significant contraction and functional shift within the S‐specific CD4 T‐cell compartment. Critically, early S‐specific T‐cell responses, particularly from Tph cells, predicted the durability of neutralising titres.

Anti‐S IgG, measured by live cell‐based binding assays, correlated strongly with neutralising titres throughout convalescence, consistent with previous findings.^20^ Both showed significant, progressive decline over 14 months after mild‐to‐moderate infection but remained detectable in most participants, aligning with studies extending to 8 months.^9^, ^12^, ^13^ As our baseline sample collection occurred later (median ~ Day 50 post infection) than studies commencing shortly after symptom onset (e.g. 25–30 days),^9^, ^18^, ^34^ our calculated antibody half‐lives were longer, reflecting the slower second decay phase. Our estimated half‐lives fall within the 200–500 days range reported for antibody persistence beyond Day 70 post infection.^12^, ^13^, ^20^

We observed a positive correlation between advancing age and higher anti‐S IgG and neutralising titres in early convalescence, which extended, for anti‐S IgG, to 12 months post infection, consistent with previous reports.^18^, ^20^ This contrasts with short‐term vaccine studies (21–30 days) in SARS‐CoV‐2 naïve individuals showing negative age associations.^35^, ^36^, ^37^ Insufficient severe/critical cases in our cohort (*n* = 5) precluded determining disease severity effects on antibody kinetics, though marked individual variability was observed within severity categories, consistent with shorter term studies.^16^

During both stages of convalescence, Th1‐biased IFNγ and IL‐2 responses were more pronounced towards the S and M proteins than the N protein, likely reflecting genuine immunodominance^30^, ^38^ rather than peptide pool size differences, which composed of 327, 60 and 104 peptides, respectively. Fluorospot analyses revealed stable S‐specific responses over 11–14 months, even when age‐stratified, aligning with existing literature looking at total T‐cell responses.^14^, ^39^, ^40^

Existing literature does show some decline when T‐cell subsets are addressed by AIM or intracellular cytokine staining assays, but these parameters have rarely been looked at together. By utilising a high‐parameter flow cytometry panel, incorporating phenotyping, AIMs and cytokine markers, we were able to gain deeper insights into S‐specific CD4 T‐cell subsets and functionality in SARS‐CoV‐2 infection, and their potential roles in promoting a robust and durable immune response. Contrasting with the stable Fluorospot data, S‐specific AIM^+^ CD4 T cells significantly declined post‐infection. Subset analysis showed reduced effector memory T cells and a relative increase in naïve‐like cells, consistent with immune response contraction. Concurrently, cTfh and Tph cell frequencies decreased over time. Tph cells exhibited pronounced polyfunctionality (simultaneous IFNγ, IL‐2, IL‐21 and TNF production), compared to their cTfh counterparts, and were the predominant producers of the B cell‐helping cytokine, IL‐21.

The prominent role of Tph cells, rather than canonical cTfh cells, in supporting antibody responses distinguishes our findings from previous paradigms and highlights a distinct adaptive pathway post‐SARS‐CoV‐2 infection. While prior work established roles for polyfunctional Th1‐like responses and cTfh cells in early viral containment and vaccine responses,^25^ and Tph cells in extrafollicular antibody responses,^6^ our high‐parameter analysis demonstrates that Tph cells—particularly highly polyfunctional and IL‐21^+^ subsets—are most closely tied to the induction and persistence of neutralising and anti‐S IgG titres. This aligns with emerging longitudinal data,^6^, ^26^, ^29^, ^41^ suggesting early Tph responses act as a critical determinant of durable humoral memory.

Mechanistically, the dominance of Tph‐derived IL‐21and their extensive cytokine polyfunctionality, suggests these cells act as versatile helpers, providing signals for B‐cell class switching and affinity maturation while contributing to the broader antiviral milieu. Persistent TNF production by S‐specific CD4^+^ T cells, even as other functions wane, is notable as TNF supports central memory maintenance and ongoing germinal centre reactions.^23^ The stronger association of Tph versus cTfh with antibody magnitude and longevity may reflect incongruence between cTfh and lymph node Tfh.^42^

Importantly, ageing also positively correlated with S‐specific Tph cell frequency and binding/neutralising antibody levels, indicating possible combined effects of host age and Tph‐driven B cell help in shaping long‐term serological immunity. This aligns with immunosenescence findings: older adults have higher frequencies of polyfunctional Tfh/Tph cells and IL‐21‐producing CD4^+^ subsets, correlating with more robust but sometimes dysregulated antibody responses.^43^, ^44^, ^45^ This age‐associated IL‐21 increase may enhance durable humoral responses; however, it also raises the possibility of age‐related immune remodelling contributing to the risk of immunopathology following infection.

Tph cells support extrafollicular atypical B cells (also termed ‘age‐associated’ B cells), reportedly elevated in COVID‐19.^6^, ^26^, ^29^, ^46^ We attempted to investigate a relationship between Tph and atypical B cells by measuring RBD^+^IgG^+^CD27^−^CD21^−^ atypical B cells using Wuhan spike RBD tetramers, but acquired events were insufficient for conclusive correlations.

### Strengths

This study demonstrated longitudinal kinetics of SARS‐CoV‐2 humoral and T‐cell immunity in a ‘clean’ cohort without reinfection or immunisation over 11–14 months, with little other circulating respiratory infections. Our detailed analysis of T‐cell responses via high‐dimensional flow cytometry, combining multiple cytokines (including IL‐21) and activation‐induced markers in the same cells, enabled correlations between antibody, S‐specific T cell subsets and functions, age and time.

### Limitations

There are important caveats to interpretation. Our cohort was limited in size, especially for severe disease (*n* = 5), and based mainly on Wuhan variant infection, the predominant variant at the time of collection. For this reason, we have only measured Wuhan‐specific responses. Additional cohorts are required to confirm these findings across other SARS‐CoV‐2 variants. Because of sample availability, detailed T cell analysis was conducted in a subset of participants (*n* = 14). The study focussed on mild and moderate disease, and whether similar Tph cell dynamics operate in severe or immunosuppressed cases remains unknown. Causality between Tph cells, IL‐21, and long‐term antibody persistence can only be inferred. While blood polyfunctional Tph cells correlated more tightly with antibody maintenance than cTfh cells post‐infection, further studies examining the function of these cells, and direct effects of polyfunctional T cells in infected tissues are needed.

## Conclusion

Overall, our findings delineate the evolving interplay between CD4^+^ T‐cell subsets and humoral immunity after SARS‐CoV‐2 infection. Early polyfunctional Tph responses are critical predictors of durable antibody levels, particularly in older adults where this population tends to be increased. Our data showed a greater role for Tph than cTfh in supporting neutralising antibody activity, which has been previously underappreciated. Continued examination of Tph functionality in breakthrough infection in vaccinees will refine strategies to bolster immune memory against current and emerging variants. This could guide vaccine and booster strategies to elicit high‐quality T cell help for sustained immunity.

## Methods

### Participants and clinical samples

Unvaccinated adults with laboratory‐confirmed SARS‐CoV‐2 infection convalescent patients were recruited from hospitals in Sydney, NSW, Australia, between 2020 and 2022 in one of two cohorts: from Westmead Hospital or by the COSIN (Collection of COVID‐19 Outbreak Samples in NSW) study^47^ in Sydney, NSW, Australia, during 2020 and 2022. In total, 67 participants were included in this analysis study, and samples were collected where possible at 1–3 months, 3–6 months, 6–9 months and 11–14 months post SARS‐CoV‐2 recovery (Table 1). All samples were collected from unvaccinated participants, and infection dates occurred prior to the implementation of SARS‐CoV‐2 antiviral medications. Their disease severity ranged from mild to critical, classified as per NIH guidelines^48^; all participants were presumed to be infected with the 2019 Wuhan variant, as no other variants had been identified in Australia at the time of infection, apart from five donors who were presumed to be infected with the Delta variant in Jul 2021. No participants were re‐infected or taking immunosuppressive medication during the study period. Blood samples were collected by venipuncture into serum‐separating tubes and lithium heparin tubes. Peripheral blood mononuclear cells (PBMC) were isolated from the latter by Ficoll density gradient centrifugation and cryopreserved.

### SARS‐CoV‐2 spike IgG assay

A flow cytometry live cell‐based assay was performed as previously described^20^, ^30^, ^40^, ^49^ to measure binding IgG against SARS‐CoV‐2 Spike (anti‐S IgG). In brief, HEK293 cells expressing SARS‐CoV‐2 spike protein (Wuhan‐1 D614) were incubated with diluted participant sera, then anti‐IgG‐AF647 (Thermo Fisher Scientific). Data were acquired on a BD Biosciences LSRII flow cytometer. The threshold for seropositivity was defined as 4SD above the delta Median Fluorescence Intensity (dMFI) of controls. Assay controls were healthy and noninflammatory neurological disorder non‐infected pre‐pandemic adults (*n* = 24).

### SARS‐CoV‐2 neutralisation assay

As previously described, virus neutralisation against the original Wuhan strain was determined from serum samples.^20^ Briefly, diluted sera samples were incubated with SARS‐CoV‐2 (‘wild‐type’ control virus (A.2.2) from clade A) for 1 h before being added to HEK293T‐ACE2 cells in the presence of NucBlue (Invitrogen) live cell nuclear stain for 72 h. Samples were imaged with InCell Analyser, and nuclei counts, a proxy for cytopathic effect were compared between convalescent sera, mock controls (defined as 100% neutralisation), and infected controls (defined as 0% neutralisation).

### IFN‐γ and IL‐2 FluoroSpot assay

Cryopreserved PBMC were thawed at 37°C in RF10 (RPMI‐1640 medium w/L‐Glutamine (Lonza) supplemented with 10% FCS and 1X penicillin–streptomycin, Thermo Fisher Scientific) and rested for approximately 18 h at 37°C and 5% CO_2_. Cell viability was assessed on a Beckman Coulter CytoFLEX flow cytometer. Dual colour FluoroSpot plates pre‐coated with anti‐IFN‐γ and anti‐IL‐2 capture antibodies (Mabtech) were blocked with RPMI‐1640 medium with 10% FCS for 1 h prior to the addition of 150–300 000 PBMC per well in RF10. Samples were stimulated with either: (1) PepTivator SARS‐CoV‐2 Prot_S Complete, (2) PepTivator SARS‐CoV‐2 Prot_N, (3) PepTivator SARS‐CoV‐2 Prot_M (all Miltenyi Biotech) and (4) 10 μg mL^−1^ PHA‐L (Abacus DX), in addition to unstimulated wells (RF10 only). All peptide pools were added at 1 μg mL^−1^. Plates were incubated at 37°C and 5% CO_2_ for 20 h before the assay was developed according to the manufacturer's instructions. Fluorescent spots indicating cells that secreted IFN‐γ (FITC filter) and/or IL‐2 (Cy‐3 filter) were detected with a Mabtech IRIS FluoroSpot reader. The mean number of responding cells in unstimulated controls was subtracted from stimulated samples to account for background, and results were expressed as spot‐forming units (SFU) per 1 × 10^6^ PBMC.

### Intracellular cytokine staining assay

Details of the antibody staining panel, which was adapted from OMIP‐060,^50^ can be found in Supplementary table 3. Cryopreserved PBMC were thawed, rested and counted as described above. PBMC (1–1.5 × 10^6^ cells) were cultured in AIM‐V medium (Thermo Fisher Scientific) with either (1) 1 μg mL^−1^ PepTivator SARS‐CoV‐2 Prot_S Complete, (2) 50 ng mL^−1^ PMA and 0.625 μM ionomycin or (3) medium for 6 h (to reduce bystander activation) in the presence of anti‐CD28 and anti‐CD49d antibodies (1 μg mL^−1^, BD Biosciences), anti‐CD107a and anti‐CXCR5. Brefeldin A (GolgiPlug 1.4 mL mL^−1^; BD Biosciences) and monensin (GolgiStop, 0.9 mL mL^−1^; BD Biosciences) were then added 1 h into the culture. Following incubation, cells were washed and stained with Fixable Viability Stain 440UV (BD Biosciences) for 20 min at RT. Cells were washed and incubated with surface antibodies for 20 min at RT (Supplementary table 3). Cells were then permeabilised for 30 min using Foxp3 Transcription Factor Fixation/Permeabilisation (Invitrogen, eBioscience), washed with Perm/Wash Buffer (BD Biosciences) and intracellular staining was performed for 30 min at RT. Cells were fixed with 1% paraformaldehyde (ProSciTech) and kept at 4°C until acquisition on a FACSymphony flow cytometer (BD Biosciences). The data were analysed using FlowJo v10; the gating strategy is shown in Supplementary figure 2. To study the polyfunctionality of the CD4 AIM^+^ T cells, Boolean gating was performed using IFN‐γ, IL‐2, TNF and IL‐21, in FlowJo and analysed using SPICE 6.1 software (https://niaid.github.io/spice/) following the technical considerations published by the software developers^42^, ^51^. To identify the functionality of the subsets, individual T1 Spike‐ stimulated samples were concatenated in FlowJo and subjected to unsupervised clustering using the FlowSOM plugin^43^, ^52^, based on phenotypic markers (CXCR5, PD‐1) and intracellular cytokines (IFNγ, IL‐2, IL‐21, TNF). Clusters were visualised and refined using the Cluster Explorer plugin in FlowJo, where clusters with similar normalised mean fluorescence intensity (MFI) patterns were merged.

### Data analysis

For the linear mixed effects (LME) modelling, variables were log‐transformed to approximate normality and to stabilise the variance prior to analysis. LME models were used to estimate within‐participant changes in antibody and FluoroSpot outcomes both by time point (T1, T2, T3 and T4) and by months from diagnosis to sample collection, and to test for evidence of interaction between these changes and age group. In these models, participant ID was the group identifier. The discrete time points, and the continuous linear and quadratic time covariates were fitted separately as both fixed and random effects with a general positive‐definite covariance matrix. The two‐way interactions between the fixed effects of time and each of the fixed categorical variables were used to assess the association between these variables. Diagnostic plots of residuals were used to check the adequacy of the fitted models. Two‐tailed tests with a significance level of 5% were used throughout. All analyses were exploratory, and there was no adjustment for multiple comparisons. The statistical software IBM SPSS Statistics version 28 and S+ Software (SolutionMetrics, Sydney) were used to analyse the data. To calculate the modelling of immune response markers, we employed a nonlinear mixed‐effects modelling approach using a one‐phase exponential decay model. Model fitting was conducted using the nlme package (version 3.1‐167) in R (version 4.4.2). Antibody levels, specifically anti‐Spike IgG and neutralising antibodies, were modelled as a function of time (days post‐infection), incorporating random effects for each participant to account for individual variability in baseline levels and decay rates. Initial parameter estimates were derived from exploratory plots and summary statistics of the observed data. The half‐life (*t*_1/2_), defined as the time required for the marker to decrease to half of its initial level, was also considered. Model diagnostics included residual analysis and visual inspection of fitted curves to assess model adequacy and goodness‐of‐fit. To evaluate the relationships between Spike‐specific CD4^+^ T cell subsets, age, days post‐infection, and humoral responses (binding IgG and neutralising antibodies), Spearman rank correlations were utilised at both early (T1: 1–3 months) and late (T4: 11–14 months) time points. The Benjamini–Hochberg false discovery rate method was applied to adjust *P*‐values for multiple comparisons^1^. Analyses and data processing were executed in R v4.4.1, with the Hmisc package facilitating Spearman calculations and corrplot aiding in matrix visualisation. Significant associations were analysed to pinpoint early T cell predictors of sustained antibody responses. Additional analyses, as described in the figure legends, were conducted on untransformed data using either GraphPad Prism 10 or R version 4.2.2.

## Author contributions

**Katie Tungatt:** Methodology; investigation; formal analysis; visualization; writing – original draft; writing – review and editing. **Gabriela Martins Costa Gomes:** Methodology; investigation; formal analysis; visualization; writing – original draft; writing – review and editing. **Nicole L Fewings:** Methodology; investigation; formal analysis; visualization; writing – original draft; writing – review and editing. **Aija Stubis:** Investigation; formal analysis; visualization; writing – review and editing. **Chloe M Doyle:** Investigation; writing – review and editing. **Vera Merheb:** Writing – review and editing. **Anupriya Aggarwal:** Writing – review and editing. **Karen Byth:** Methodology; formal analysis; writing – review and editing. **Harry Robertson:** Formal analysis; writing – review and editing. **Suat Dervish:** Methodology; writing – review and editing. **Susan Maddocks:** Resources; writing – review and editing. **Janette Taylor:** Investigation; writing – review and editing. **Rowena A Bull:** Resources; writing – review and editing. **Marianne Martinello:** Resources; writing – review and editing. **Fabienne Brilot:** Methodology; investigation; writing – review and editing. **Stuart G Turville:** Methodology; investigation; writing – review and editing. **Anthony L Cunningham:** Conceptualization; methodology; visualization; funding acquisition; supervision; writing – original draft; writing – review and editing. **Kerrie J Sandgren:** Conceptualization; methodology; investigation; formal analysis; visualization; funding acquisition; supervision; writing – original draft; writing – review and editing.

## Conflict of interest

The authors declare no conflict of interest.

## Study approval

The study protocol was approved by the Human Research Ethics Committees of the Western Sydney Local Health District, NSW Australia (2020/ETH00844) and was conducted according to the Declaration of Helsinki and International Conference on Harmonization Good Clinical Practice (ICH/GCP) guidelines and local regulatory requirements. Written informed consent was obtained from all participants before study procedures. Additional participant samples were obtained from the COSIN study, approved by the Human Research Ethics Committees of the Northern Sydney Local Health District and the University of New South Wales, NSW Australia (ETH00520).

## Supporting information


Supplementary data 1

## Data Availability

The data that support the findings of this study are available from the corresponding author upon reasonable request.
