# Supplementary material for: Polyfunctional T peripheral helper cells are associated with the magnitude and durability of antibody responses after COVID‐19
Source: Clin Transl Immunology. 2025 Dec 18;14(12):e70070. doi: 10.1002/cti2.70070 (PMC12712866; doi:10.1002/cti2.70070)
Supplement: Supplementary file 1 — Supplementary data 1 [file CTI2-14-e70070-s001.docx]

**Supplementary Figures & Tables**


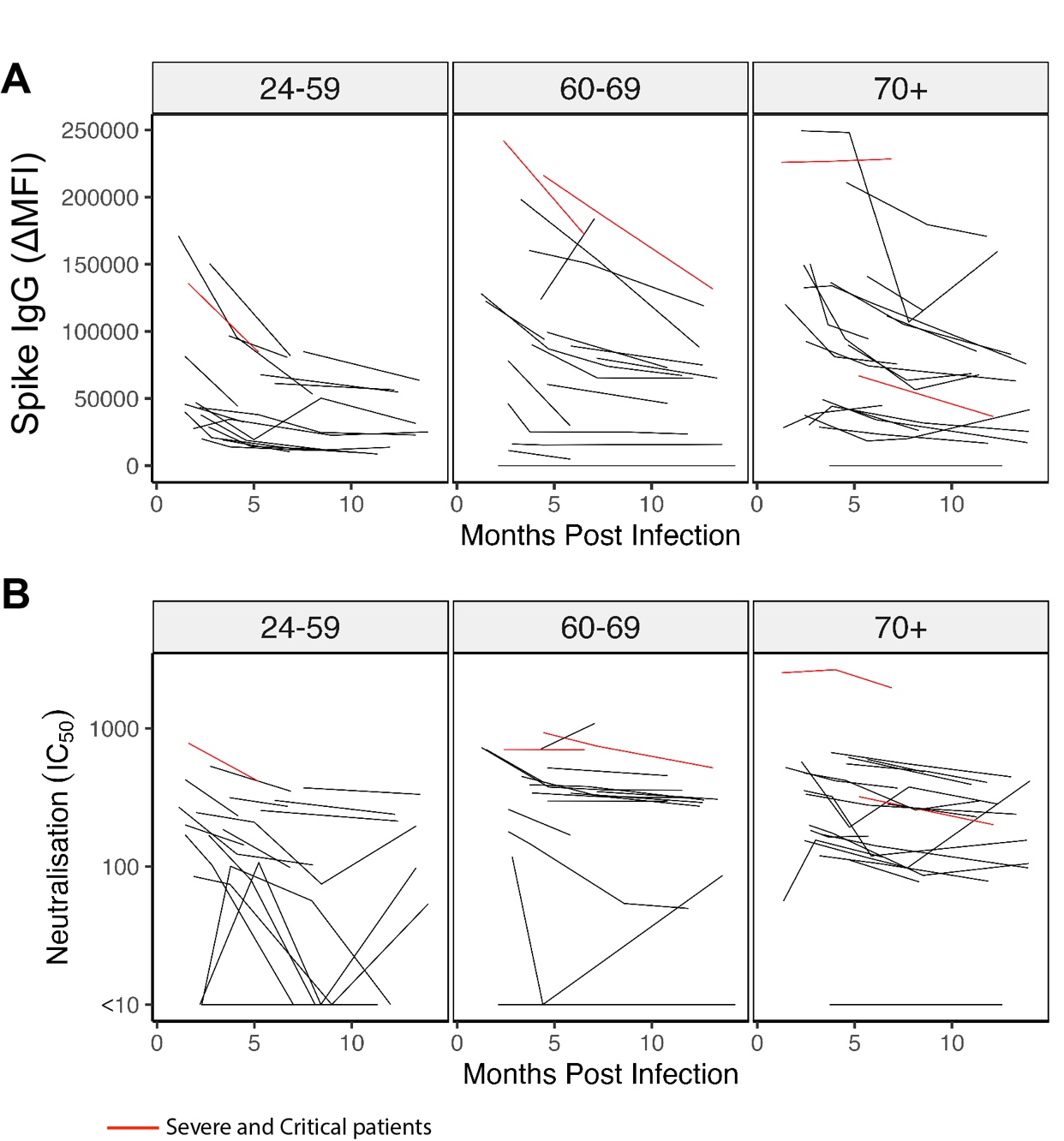


**Supplementary Figure 1: Anti-SARS-CoV-2 spike IgG and neutralising antibody responses by age on a continuous time scale post SARS-CoV-2 infection.**

**A)** Anti spike IgG (measured in delta median fluorescence intensity (∆MFI)) and **B)** virus neutralisation against the original Wuhan strain were measured from 1 to 14 months post infection. The participant data has been divided between three age groups, 20-59, 60-69 and ≥ 70 years old at the time of infection. Each line represents one participant and at least two timepoints collected, up to four. Red lines denote participants who were categorised as severe or critical for disease severity. Linear mixed effects modelling was performed on log transformed data and is reported in Supplementary Table 1.

**Supplementary Table 1: The effect of time and age on antibody and T-cell responses (p-values from Linear Mixed Effects Models)**

| **Variable*** | | **Time** ^#^ | | **Time** ^#+^ **x Age Group** |
| --- | --- | --- | --- | --- |
|  |  | **Linear** ^+^ | **Quadratic ^$^** |  |
| Anti-SARS-CoV-2 spike IgG antibody | | <0.001 | 0.001 | 0.897 |
| SARS-CoV-2 Wuhan neutralising antibody | | <0.001 | 0.001 | 0.819 |
| Response to spike | IFN-γ | 0.783 | NS | 0.136 |
|  | IL-2 | 0.832 | NS | 0.491 |
|  | IFN-γ and IL-2 | 0.685 | NS | 0.648 |
| Response to nucleocapsid | IFN-γ | 0.435 | NS | 0.736 |
|  | IL-2 | 0.861 | NS | 0.623 |
|  | IFN-γ and IL-2 | 0.983 | NS | 0.867 |
| Response to membrane | IFN-γ | 0.736 | NS | 0.110 |
|  | IL-2 | 0.329 | NS | 0.437 |
|  | IFN-γ and IL-2 | 0.445 | NS | 0.230 |

*^*^ All analyses done on log_10_ transformed variables*

*^#^ Continuous time from diagnosis to sample collection*

^+^ *Continuous linear time covariate*

*^$^ Continuous quadratic time covariate*

*p-values that met the significance level of 5% are highlighted*

**Supplementary Table 2: T cell ICS/AIMs flow cytometry cohort**

|  | | **24 – 59**  6 (42.9%) | **60 – 69**  3 (21.4%) | **70+**  5 (35.7%) | **Total cohort**  14 |
| --- | --- | --- | --- | --- | --- |
| **Sex** | **Male** | 4 (66.7%) | 1 (33.3%) | 2 (40%) | 7 (50%) |
|  | **Female** | 2 (33.3%) | 2 (66.7%) | 3 (60%) | 7 (50%) |
| **Disease severity** | **Mild** | 3 (50%) | 1 (33.3%) | 5 (100%) | 9 (64.3%) |
|  | **Moderate** | 3 (50%) | 2 (66.7%) | 0 (0%) | 5 (35.7%) |

**Supplementary Table 3: Antibody Panel**

| **Antibody** | **Fluorophore** | **Clone** | **Detector** | **Host Species** | **Source** | **Working dilution** |
| --- | --- | --- | --- | --- | --- | --- |
| CXCR5 ^*^ | BB515 | RF8B2 | B515_30 | Rat | BD Bioscience | 1:20 |
| IFN-γ ^†^ | BB700 | B27 | B710_50 | Mouse | BD Bioscience | 1:66 |
| IL-21 ^†^ | AF647 | 3A3-N2.1 | R670_30 | Mouse | BD Bioscience | 1:10 |
| Granzyme B ^†^ | R718 | GB11 | R710_40 | Mouse | BD Bioscience | 1:80 |
| CD38 | APC-Fire810 | SI7015A | R820_60 | Mouse | Biolegend | 1:20 |
| CCR7 | BUV395 | 2-L1-A | U379_28 | Mouse | BD Bioscience | 1:10 |
| Fixable Viability Stain 440UV | N/A | N/A | U450_50 | N/A | BD Bioscience | 1:780 |
| CD4 ^†^ | BUV496 | SK3 | U515_30 | Mouse | BD Bioscience | 1:20 |
| CD3 ^†^ | BUV563 | UCHT1 | U586_15 | Mouse | BD Bioscience | 1:20 |
| CD25 | BUV615 | 2A3 | U610_20 | Mouse | BD Bioscience | 1:160 |
| CD39 | BUV661 | TU66 | U670_30 | Mouse | BD Bioscience | 1:80 |
| CD95 | BUV737 | DX2 | U740_35 | Mouse | BD Bioscience | 1:40 |
| CD8 ^†^ | BUV805 | SK1 | U820_60 | Mouse | BD Bioscience | 1:160 |
| IL-2 ^†^ | BV421 | MQ1-17H12 | V427_25 | Rat | BD Bioscience | 1:40 |
| CD154/CD40L ^†^ | BV480 | TRAP1 | V474_25 | Mouse | BD Bioscience | 1:333 |
| CD45RO | BV570 | UCHL1 | V586_15 | Mouse | Biolegend | 1:20 |
| IL-17A ^†^ | BV605 | BL168 | V610_20 | Mouse | Biolegend | 1:40 |
| Ki67 ^†^ | BV650 | B56 | V677_20 | Mouse | BD Bioscience | 1:40 |
| CD69 ^†^ | BV711 | FN50 | V710_50 | Mouse | BD Bioscience | 1:80 |
| TNF ^†^ | BV750 | MAb11 | V750_30 | Mouse | BD Bioscience | 1:40 |
| CD27 ^†^ | BV786 | L128 | V820_60 | Mouse | BD Bioscience | 1:160 |
| Perforin ^†^ | PE | B-D48 | Y586_15 | Mouse | Biolegend | 1:160 |
| PD-1 | PE DAZZLE594 | NAT105 | Y610_20 | Mouse | Biolegend | 1:40 |
| CD107a ^*^ | PECY5 | H4A3 | Y670_30 | Mouse | BD Bioscience | 1:40 |
| FOXP3 † | PECY5.5 | PCH101 | Y710_50 | Rat | Invitrogen | 1:80 |
| IL-22 ^†^ | PE-CY7 | 22URTI | Y820_60 | Mouse | Invitrogen | 1:80 |

** Stained in culture*

*† Stained Intracellularly*


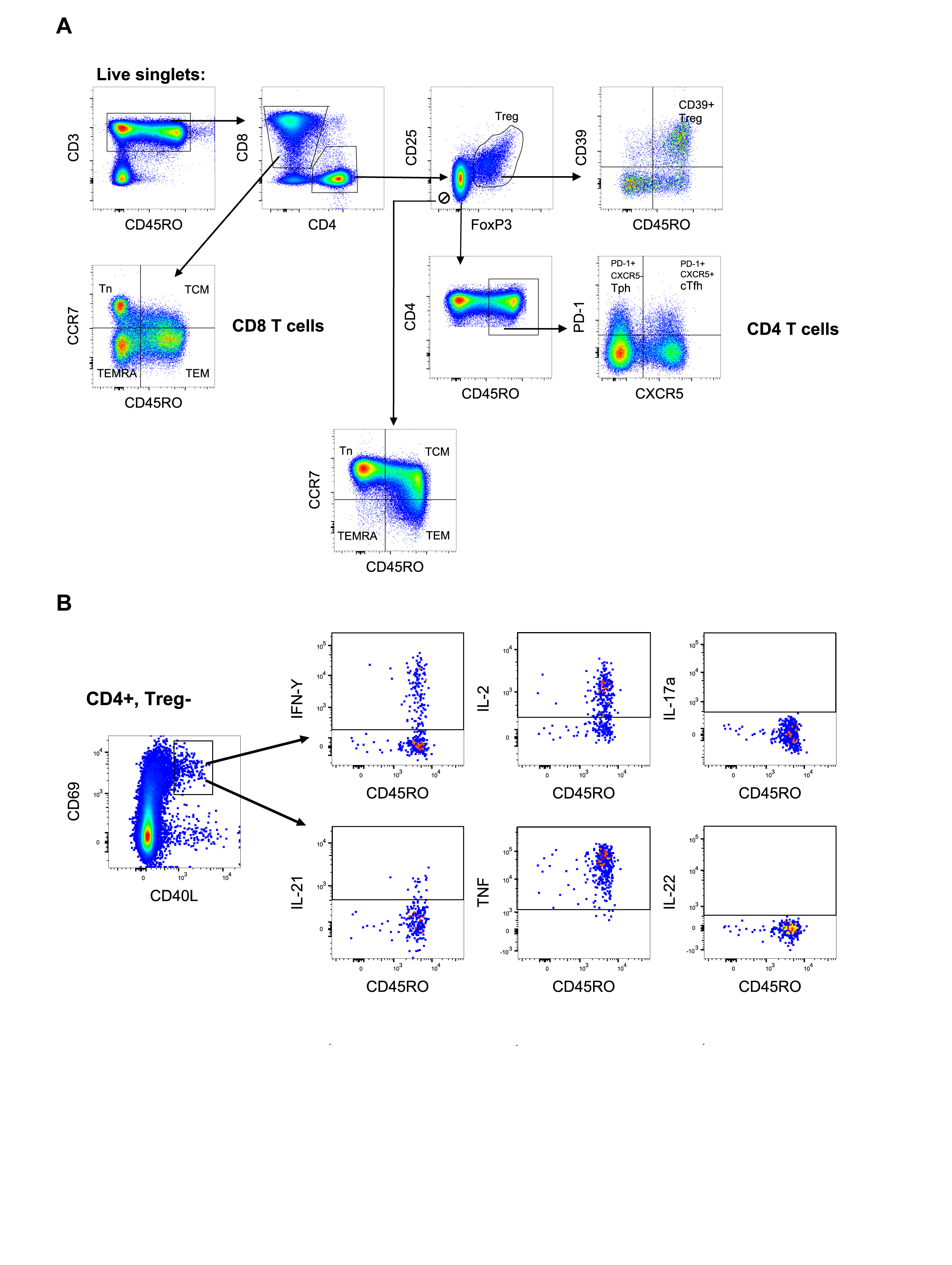


**Supplementary Figure 2: Flow cytometry gating strategy**

Flow cytometry gating strategy to identify T cell subsets, and AIM and cytokine expression. A) Amongst CD3+ CD4+ T cells, CD25^hi^FoxP3+ regulatory T cells (Treg) were classified as naïve (CD45RO-) CD39- Tregs or mature (CD45RO+) Tregs with or without CD39 expression. Circulating T follicular helper cells (cTfh) and T peripheral helper cells (Tph) were gated from non-Tregs and CD45RO+ then gated on CXCR5+, PD-1+ (cTfh) or CXCR5-, PD-1+ (Tph). Non-Tregs as well as CD8+ T cells were then gated into naïve (Tn), central memory (TCM), effector memory (TEM) and effector memory re-expressing CD45RA (TEMRA) T cells.

B) CD4 AIM+ T cells were identified from the CD4+, Treg- gate based on activation-induced markers (AIM) CD69 and CD40L expression. Cytokine expression (IFN-γ, IL-2, IL-17a, IL-21, TNF, and IL-22) was then plotted against CD45RO to assess whether the majority of cytokine-producing cells belong to the memory (CD45RO+) population or if there are a few cytokine-positive cells within the CD45RO- subset.


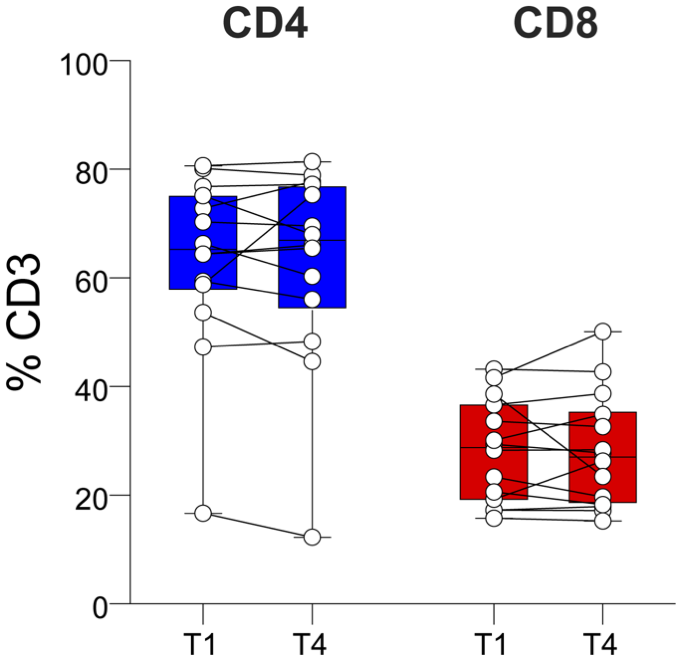


**Supplementary Figure 3: Proportions of CD4 and CD8 T cells of total CD3+ T cells post SARS-CoV-2 infection.** Samples collected at T1 (1-3 months) and T4 (11-14 months) post infection. Statistical significance was assessed using the Wilcoxon matched-pairs signed rank test. *p<0.05, ** p≤0.01, ***p≤0.001


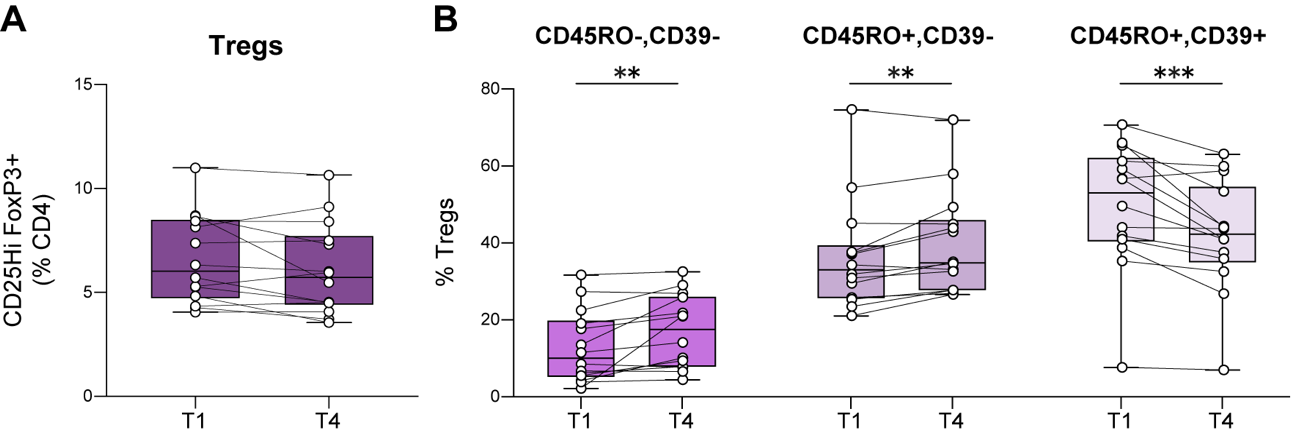


**Supplementary Figure 4: Tregs and their subsets post SARS-CoV-2 infection.** Treg cell subsets were analysed from samples collected at T1 (1-3 months) and T4 (11-14 months) post-infection. **A)** T regulatory cells (Tregs, CD25^hi^, FoxP3+) as a percentage of CD4 T cells. **B)** Proportions of Treg subsets as a percentage of total Tregs: naïve (CD45RO-, CD39-), CD39- and CD39+ mature (CD45RO+) Tregs. Statistical significance was assessed using the Wilcoxon matched-pairs signed rank test. *p<0.05, ** p≤0.01, ***p≤0.001.


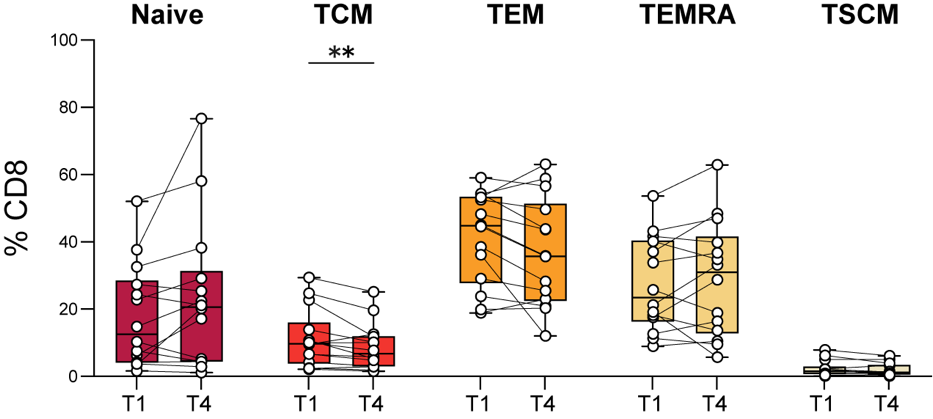


**Supplementary Figure 5: Proportions of CD8 cell subsets over time post SARS-CoV-2 infection.** Naïve and memory subsets of CD8 T cells: Central memory (TCM), effector memory (TEM), effector memory T cells re-expressing CD45RA (TEMRA), post SARS-CoV-2 infection at two time points: 1-3 months (T1) and 11-14 months (T4) post-infection.. Statistical significance was assessed using the Wilcoxon matched-pairs signed rank test. *p<0.05, ** p≤0.01, ***p≤0.001.


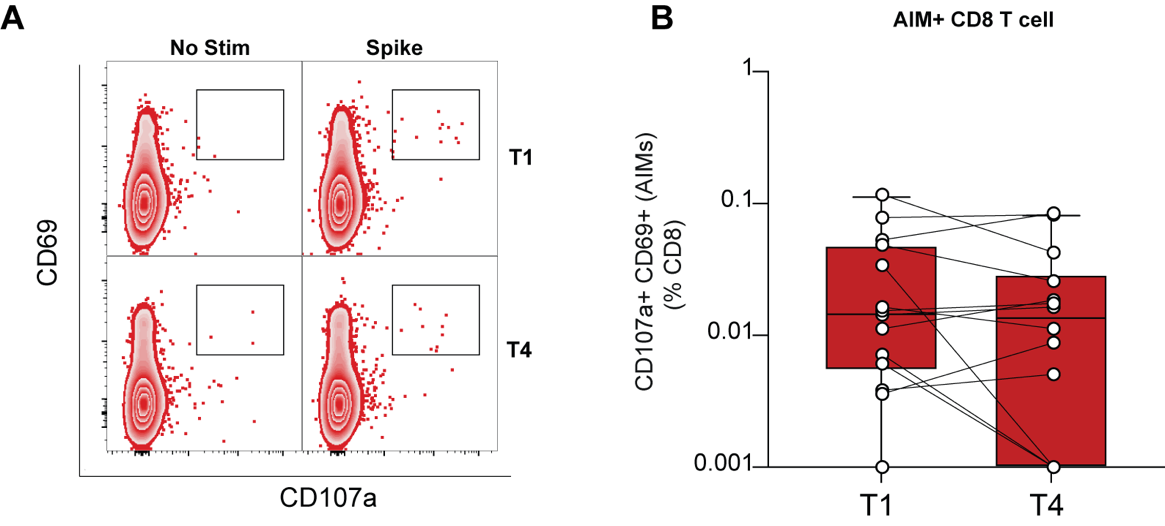


**Supplementary Figure 6: Frequency of spike-specific CD8 T cells over time post SARS-CoV-2 infection.** Subsets were analysed from samples collected at T1 (1-3 months) and T4 (11-14 months) post-infection**. A)** Representative flow cytometry plots of AIM+ SARS-CoV-2-specific CD8 T cells (CD107a+ CD69+ CD8 T cells) after 6 h stimulation with spike peptide pool compared to no stimulation. **B)** Frequency of AIM+ CD8 T cells (percentage of CD8+ T cells), values shown are background subtracted. Statistical significance was assessed using the Wilcoxon matched-pairs signed rank test. *p<0.05, ** p≤0.01, ***p≤0.001


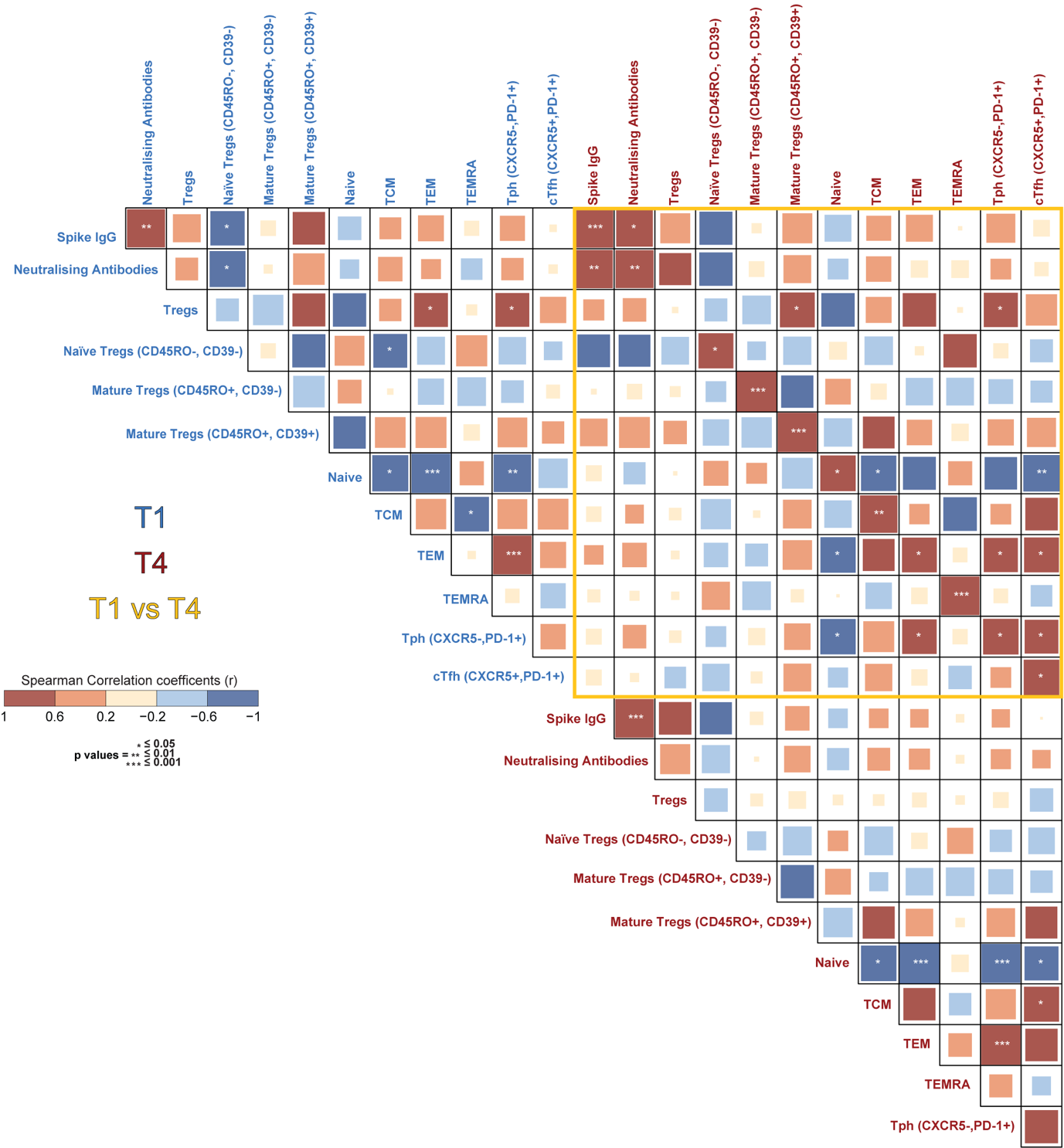


**Supplementary Figure 7: Association between antibodies and CD4 T cell subsets at different timepoints post SARS-CoV-2 infection.**  Correlation matrix heat map of multiple parameters at T1 (1-3 months) and T4 (11-14 months) post infection. Spearman’s rank correlation coefficients (r) are depicted by colour intensity, ranging from blue (negative correlation, r=−1.0) to red (positive correlation, r=1.0), and the size of the squares. Statistical significance (p-values adjusted for multiple comparisons using the Benjamini-Hochberg (BH) method is indicated by asterisks: *p<0.05, **p≤0.01, ***p≤0.001. Colour of text indicates timepoint blue = T1; red = T4. Yellow border shows T1 vs T4 correlations.
